# Prognostic role of CRABP2 in lung cancer: a meta-analysis

**DOI:** 10.1186/s13019-024-02887-5

**Published:** 2024-06-24

**Authors:** Guang Yang, Qifan Yin, Wenhao Wang, Siwei Xu, Huining Liu

**Affiliations:** 1 Department of Thoracic Surgery, The First Hospital of Hebei Medical University, No. 89 Donggang Street, Yuhua District, Shijiazhuang, 050031 Hebei Province People’s Republic of China; 2 Department of Thoracic Surgery, Hebei Provincal General Hospital, No.348,West He-Ping Road, XinHua District, Shijiazhuang, 050051 Hebei Province People’s Republic of China

**Keywords:** Lung cancer, Prognosis, CRABP2, Meta-analysis

## Abstract

**Background:**

The prognostic value of cellular retinoic acid-binding protein 2 (CRABP2), in lung cancer patients remains to be uncertained. Therefore, our research attempted to assess the relationship between CRABP2 and survival analysis in lung cancer patients through meta-analysis.

**Method:**

Related literature retrieved from Cochrane Library, Ovid, Embase, PubMed, the CNKI, and the Web of Science. The latest update of the search was May 1, 2023. The outcome indicators included as effective measures in the study were hazard ratio (HR), and 95% confidence interval (CI). The Stata 12.0 software was used to analyze the data.

**Results:**

A total of4 studies were finally enrolled in our meta-analysis. The increased plasma level of CRABP2 predicted poor OS in lung cancer patient with a combined HR of 1.14 (95% CI: 1.00–1.30), and were not associated with poor PFS with combined HR: 1.15% CI: 0.63–2.09) in lung cancer patients.

**Conclusions:**

Our meta-analysis found the increased plasma level of CRABP2 was associated with poor OS independently in NSCLC patients. The plasma CRABP2 level may be an indicator of biological aggressiveness of the tumor. Our research was promising regarding the feasibility and utility of plasma CRABP2 as a novel prognostic biomarker in NSCLC, and the findings warrant further investigation.

## Introduction

Lung cancer is the leading cause of cancer related deaths worldwide, which has posed a serious threat to the health and lives of people across the world [1]. According to histological subgroups, lung cancer is divided into two large categories: non-small cell lung cancer (NSCLC) and small cell lung cancer (SCLC), with NSCLC contributing to around 85% of all cases [2]. Despite recent advances in diagnostic and therapeutic procedures, including development of computed tomography–based screening to detect lung cancer early, molecular-targeted therapy and immunotherapy in the advanced lung cancer treatment, The prognosis of lung cancer patients remains poor, with a 5-year overall survival rate of 16% [3–5]. Research efforts have focused on identifying new biomarkers and molecular pathways that influence NSCLC progression. The recent discovery of CRABP2 and the exploration of the associated mechanisms have resulted in great advances in the area of lung cancer [6, 7]. Studies have shown plasma CRABP2 levels might be a novel and promising prognostic marker in NSCLC.

Retinoic acid (RA) is the most active metabolite of vitamin A and could influence the proliferation, differentiation, and apoptosis of normal and tumor cells [8, 9]. A particular carrier for RA is cellular retinoic acid-binding protein 2 (CRABP2), a small cytosolic protein from the family of intracellular lipid-binding proteins [10]. By transiting RA to the nucleus and interacting with the nuclear retinoic acid receptor (RAR), CRABP2 activates RA’s transcriptional regulatory function and has the biological effects of controlling cell proliferation, death, and metastasis [11–13]. Some studies revealed aberrant CRABP expression in human cancers, which can be associated with clinicopathological characteristics and clinical prognosis. Thus, the decrease of CRABP2 expression was significantly associated with the presence of distant lymph node metastases in esophageal squamous cell carcinoma [14]. In addition, hypermethylation of CRABP2 promoter had an unfavorable prognostic value for hepatocellular carcinoma patients [11]. Conversely, Studies have reported that the absence of CRABP2 protein was associated with poor prognosis for head and neck squamous cell carcinoma patients [15]. On the other hand, the increase of CRABP2 expression was observed in later stages of human breast cancer [16]. Furthermore, in non-small cell lung cancer, kidney cancer, and glioma, elevated expression of CRABP2 was substantially related to a poor tumor prognosis [6, 17, 18]. These results indicate that CRABP2 may be involved in tumor growth and have controversial functions in various tumor types. The prognostic value of CRABP2 in lung cancer patients is still unknown. As a result, we intend to conduct a meta-analysis to comprehensively and systematically understand the prognostic value of CRABP2 in lung cancer patients.

This study investigated the prognostic role of CRABP2 in lung cancer from the perspective of 2 survival indicators: OS and PFS. OS refers to the time from the beginning of the study until to death (due to any reason). PFS refers to the time from the beginning of the study until to the progression of the disease (due to any reason). Currently, only a few studies are available on the role of CRABP2 in lung cancer. The findings from these studies were taken into consideration in order to outline the criteria for exploration and explanation of the connection between CRABP2 and its prognostic value in lung cancer patients. The present study was conducted to assess the prognostic value of CRABP2 in lung cancer.

## Material and methods

### Search strategy

The following databases were searched for the retrieval of relevant data: Cochrane Library, Embase, PubMed, and the Web of Science. The latest update of the search was May 1, 2023. The key terms used in the searches included “CRABP2” (e.g., “cellular retinoic acid-binding protein 2”); “Cellular Retinoic Acid-binding Protein 2” (e.g., “Crabp2”); and “lung cancer”[e.g., “lung neoplasm”, “lung carcinoma”,“non-small cell lung cancer (NSCLC)”,“small cell lung cancer (SCLC)”].

### Inclusion and exclusion criteria

Inclusion criteria for selecting the studies for this meta analysis were as follows criteria: (1) studied patients with lung cancer were pathological examination confirmed; (2) the connection of CRABP2 with OS and/or progression-free survival (PFS) was reported. The exclusion criteria included letters, abstracts, reviews, case reports or basic studies; research reports of studies in languages other than Chinese and English; studies lacking data on assessing hazard ratio (HR), and 95% confidence interval (CI); and studies had duplicated data or analysis.

### Data extraction and quality assessment

Two investigators (Guang Yang and Wenhao Wang) were involved in collecting the data. They independently procured the data from the available studies. In case where the retrieved studies could not be classified by title and abstract, the full text was reviewed. If disagreement appeared, two investigators discussed and reached consensus with a third investigator (Siwei Xu). During the data review process, details with respect to the following points were recorded: first author; year of publication; nationality; type of cancer; plasma level of CRABP2 expression; sample size; outcome indicators; HRs with 95% CIs. The Newcastle–Ottawa Scale (NOS) was used to assess each of the included studies quality by two independent authors (Guang Yang and Wenhao Wang). The NOS consists of three parts: selection (0-4points), comparability (0–2 points), and outcome assessment (0–3 points).The studies with NOS scores of ≥ 6 were regarded as high-quality studies and eventually were enrolled into our meta-analysis.

## Statistical analysis

All statistical analyses were conducted using Review Manager 5.3(The Nordic Cochrane Centre, The Cochrane Collaboration, Copenhagen, Denmark) and STATA 12.0 software (STATA, College Station, TX). We directly obtained HR and 95%CI from each literature or estimated these data, HR > 1 indicated a worse prognosis in lung cancer patients with the elevated plasma level of CRABP2. Cochran’s Q test and Higgins I-squared statistic were undertaken to assess the heterogeneity of the included trials. Both fixed-effects (Mantel–Haenszel method) and random effects (DerSimonian—Laird method) models were used to calculate the pooled HRs and 95%CIs. A Pheterogeneity^2^ > 50% suggested significant heterogeneity in the literature and a random-effect model was used. Otherwise, the fixed-effects model was adopted. Publication bias was assessed by Begg’s funnel plot and Egger’s linear regression test. All *P*-values were two-sided. The *P* < 0.05 was considered statistical significant.

## Results

### Identification of relevant studies

**Fig. 1:**
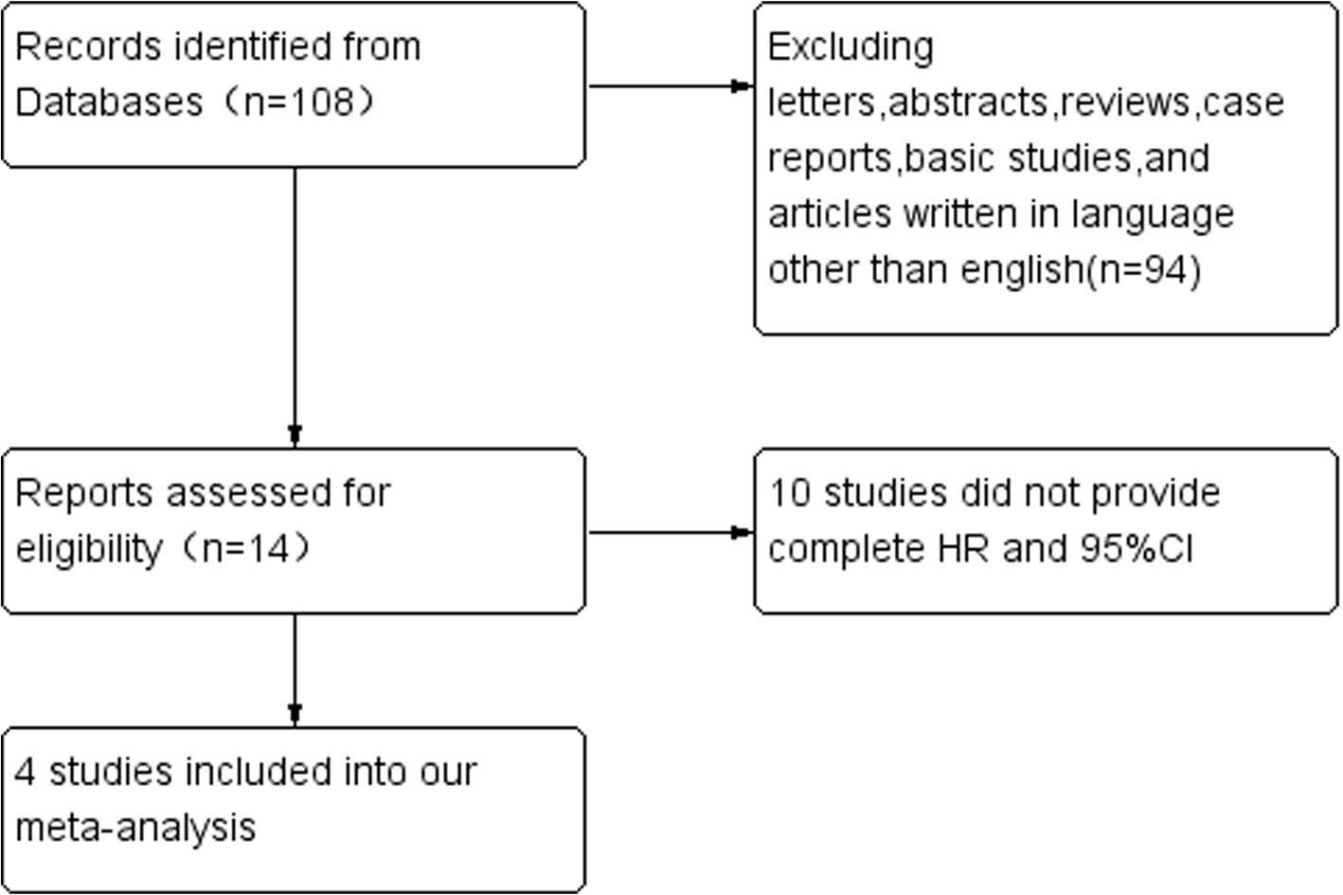
The flow diagram

A total of 14 studies published between 2013 and 2022 were initially collected. After carefully inspection of these available articles, 10 articles were eliminated and 4 studies were finally enrolled in our meta-analysis. The specific process of research selection was shown in the flow diagram (Fig. 1). Among them, two studies were from China, one study was performed in Chinese TaiWan and Korea, respectively. HRs and 95%CIs were extracted directly in 4 studies, all of which calculated HRs by multivariate analysis. In our meta analysis, 4 studies illuminated the association between plasma level of CRABP2 and OS. 2 studies reported the association between plasma level of CRABP2and PFS. The characteristics of the enrolled studies were shown in Table 1:

**Table 1:** The basic characteristics of enrolled studies

First author	Year	Region	Indicator	Outcome (HR and 95% CI)	NOS
Do Jun Kim	2018	Korea	OS	1.01 (1.00–1.02)	7
Yong Zhang	2015	China	OS	1.3 (0.98–1.73)	8
Shuangshuang Zeng	2023	China	OS	1.2 (1.04–1.38)	8
			PFS	0.84 (0.64–1.09)	
Jun-I Wu	2018	Chinese TaiWan	OS	1.2 (1.04–1.39)	7
			PFS	1.55 (1.26–1.9)	

### The plasma level of CRABP2 and OS in lung *cancer*

**Fig. 2:**
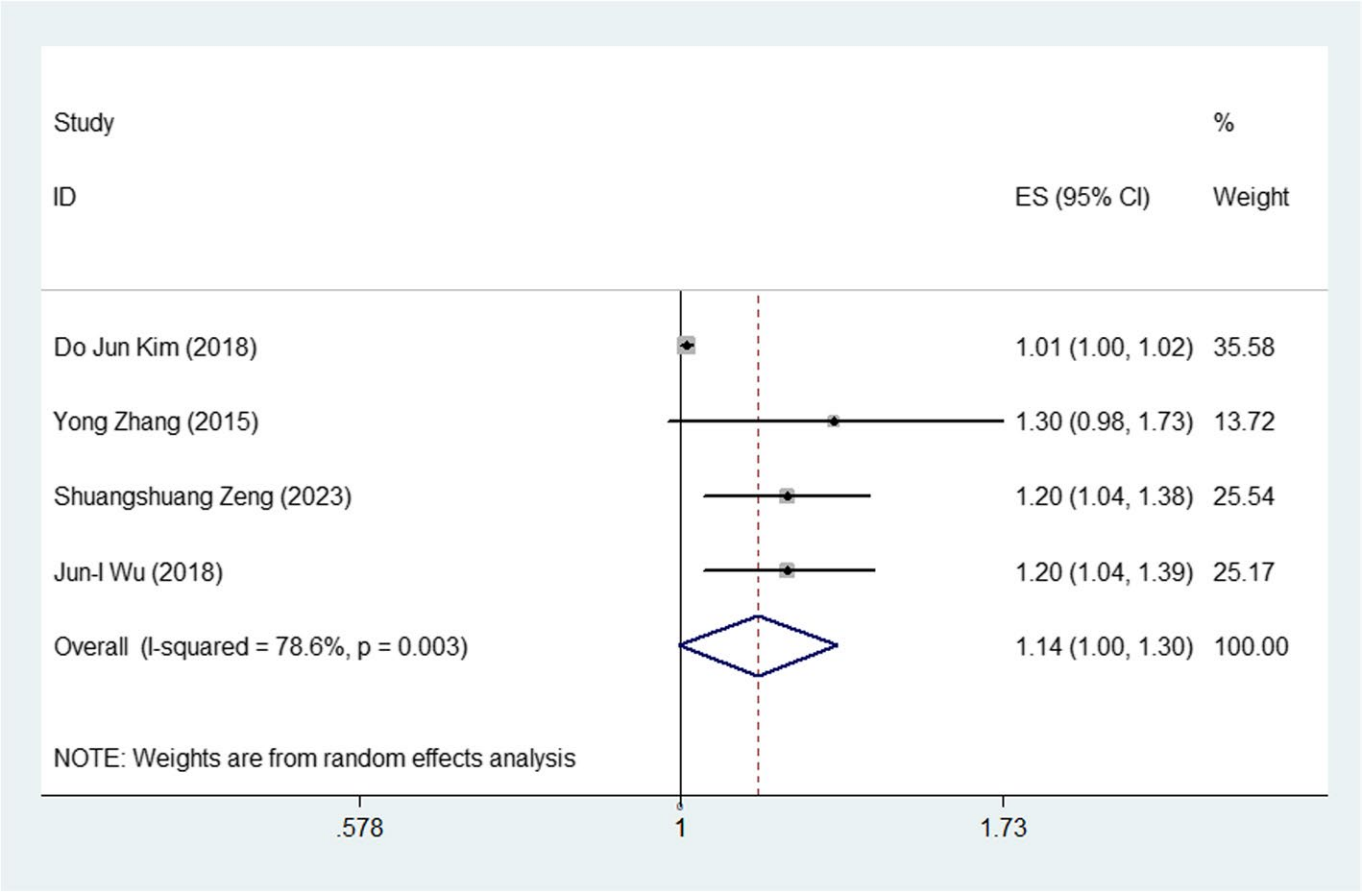
Meta-analysis of the relation between the plasma level of CRABP2 and OS in lung cancer

Four studies reported the association between the elevated plasma level of CRABP2 and OS in lung cancer. For the purpose of analyzing the indicator of OS, 4 studies were selected. Since the obvious heterogeneity was observed in the studies (I^2^ = 78.6%, *P* = 0.003), random effects model was adopted. The obtained results suggested that increased plasma level of CRABP2 predicted poor OS in lung cancer patient with a combined HR of1.14 (95% CI: 1.00–1.30; Fig. 2).

### The plasma level of CRABP2 and PFS in lung *cancer*

**Fig. 3:**
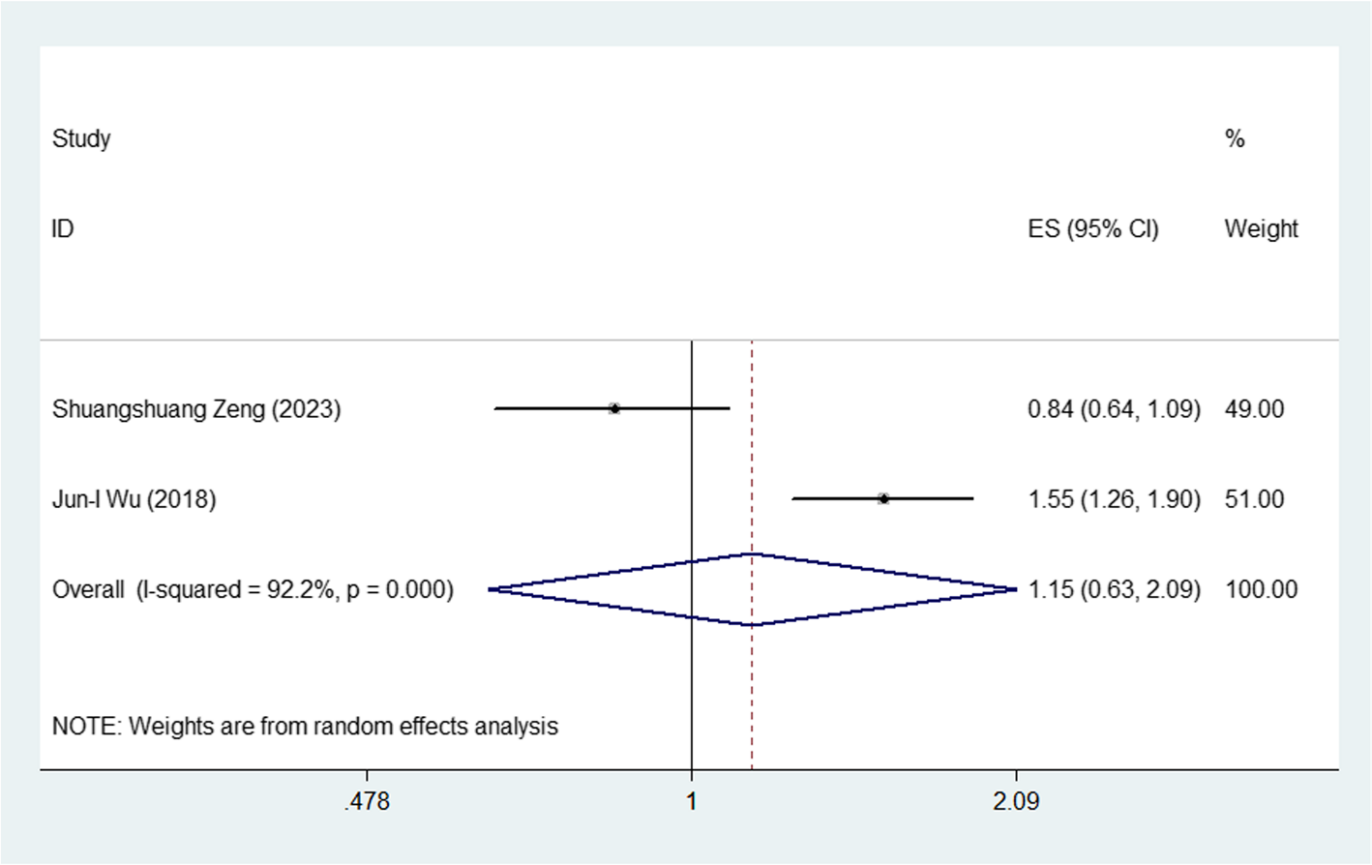
Meta-analysis of the association between the plasma level of CRABP2 and PFS in lung cancer

Two studies illustrated the association between the elevated plasma level of CRABP2 and PFS in lung cancer. Since the obvious heterogeneity was observed in the studies (I^2^ = 92.2%, *P* = 0.0001), random effects model was adopted. Meta-analysis of these two studies revealed that patients with the increased plasma level of CRABP2 were not associated with poor PFS with combined HR: 1.15% CI: 0.63–2.09, Fig. 3) in lung cancer.

### Publication *bias*

**Fig. 4:**
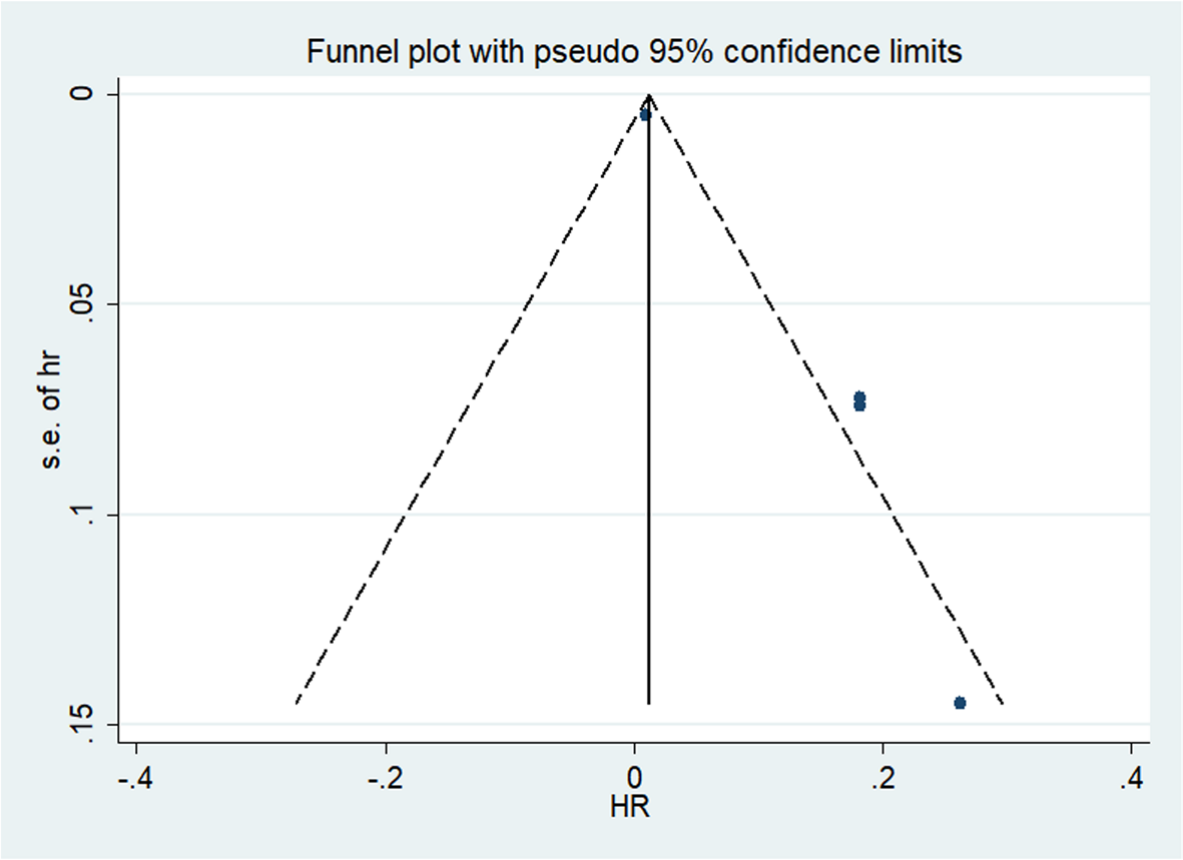
The funnel plot

Publication bias was assessed by Begg’s funnel plot and Egger’s linear regression test, No publication bias was detected for OS and PFS in Begg test (Pr > jzj = 0.764) and Egger’s test (P > jt j = 0.328). The picture of publication bias was shown in Fig. 4.

## Discussion

Our meta-analysis included 4 studies, indicating that the elevated plasma level of CRABP2 significantly predicted poor OS (HR: 1.14 (95% CI: 1.00–1.30, Fig. 1) in lung cancer patients. There were 2 studies showing the data of association between the CRABP2 and PFS in lung cancer patients. The pooled HR of 1.15 (95% CI: 0.63–2.09, Fig. 2) showed that patients with the increased plasma level of CRABP2 were not associated with poor PFS in lung cancer. Taking all these into consideration, the presence of elevated CRABP2 expression in lung cancer patients may be have a significant risk factor for poor prognostic factor for overall survival.

Lung cancer causes more than one-fourth of all cancer-related deaths worldwide [3]. Nearly sixty percent of lung cancer patients are diagnosed at late stages with metastasis, and their 5-year survival is less than 16% [19]. Thus, identifications of novel therapeutic targets against lung cancer are urgently demanded to improve patients’ survival. Cellular retinoic acid-binding proteins, Crabp1 and Crabp2, are small cytosolic proteins that belong to a family of two isotypes [10]. CRABP2is a cytosol-to-nuclear shuttling protein, which facilitates RA binding to its cognate receptor complex and transfer to the nucleus. CRABP2 encodes a member of the retinoic acid, which binds protein family and lipocalin/ cytosolic fatty-acid binding protein family [20]. CRABP2 was found to be upregulated in thyroid carcinoma and promoted the invasion, migration in thyroid cancer cells [21]. Downregulation of CRABP2 inhibits proliferation and metastasis and promotes cell apoptosis of hepatocellular carcinoma [11]. The overexpression of CRABP2 has been reported in tumor tissues of non-small cell lung cancer (NSCLC) [12, 22, 23]. However, the role of CRABP2 in lung cancer is still unclear.

Studies have shown that expression of E-cadherin increased in the empty vector group compared to the CRABP2 overexpression group. Expression of MMP9 and vimentin in the CRABP2 overexpression group was higher than that in the empty vector group and siRNA transfection group. Inhibition of CRABP2 expression could reduce cell EMT. This indicates that overexpression of CRABP2 facilitates cellular proliferation and migration, whereas CRABP2 down-regulation reduces cellular proliferation and expression of EMT-related proteins. This suggests that reducing expression of the CRABP2 gene will benefit the survival rate in patients with NSCLC [23]. Recently proposed was an antitumorigenic mechanism of CRABP2 that is independent of retinoic acid [24].

CRABP2 enhances retinoic acid signaling, which is generally considered to be an antitumor activity. However, contradictory reports exist regarding the exact role of CRABP2 in general tumorigenesis; thus, further investigation is needed [25]. Recent proteomic analyses determined that high levels of CRABP2 are an adverse prognostic marker in estrogen receptor–negative breast tumors, and another study identified CRABP2 to be a subtype-specific biomarker for ovarian cancer [26]. Expression in serous ovarian cancer specimens was up-regulated, and CRABP2 expression was positively correlated with tumor grade and cancer stage. Elevated CRABP2 mRNA transcript levels were observed in retinoblastoma tissue compared with normal retinal tissue, and infiltrating retinoblastoma showed elevated levels of CRABP2 [27]. Based on our results, this meta-analysis shown that the elevated CRABP2 expression significantly predicted poor OS in patients suffering from lung cancer. The plasma level of CRABP2 was not associated with PFS in patients with lung cancer. In conclusion, plasma CRABP2 can be used as a diagnostic and prognostic marker for NSCLC to reduce cancer cell proliferation, migration, and invasion by targeting inhibition.

Nevertheless, the meta-analysis has also posed some limitations that need to be mentioned. First, the study was retrospective in nature, which could easily lead to some bias. Second, our meta-analysis only enrolled studies published in English language. So,it was more likely to appear publication bias. Third, only a limited number of documents were selected for the purpose of analysis. Further large-scale studies and randomized controlled trials need to be conducted in the future.

## In conclusion

Our meta-analysis found the increased plasma level of CRABP2 was associated with poor OS independently in NSCLC patients. The plasma CRABP2 level may be an indicator of biological aggressiveness of the tumor. Our research was promising regarding the feasibility and utility of plasma CRABP2 as a novel prognostic biomarker in NSCLC, and the findings warrant further investigation.

## Data Availability

The datasets used and analyzed during the current study are available from the corresponding author on reasonable request.

## Abbreviations

CI: Confidence interval
PFS: Progression-free survival
HR: Hazard ratio
OS: Overall survival
CRABP2: Cellular retinoic acid-binding protein 2
NSCLC: Non-small cell lung cancer
SCLC: Small cell lung cancer
NOS: Newcastle–Ottawa Scale
RA: Retinoic acid
RAR: Retinoic acid receptor
